# Directed Evolution of Herbicide Biosensors in a Fluorescence-Activated Cell-Sorting-Compatible Yeast Two-Hybrid Platform

**DOI:** 10.1021/acssynbio.2c00297

**Published:** 2022-08-03

**Authors:** Gil Zimran, Erez Feuer, Oded Pri-Tal, Michal Shpilman, Assaf Mosquna

**Affiliations:** The Robert H. Smith Institute of Plant Sciences and Genetics in Agriculture, The Hebrew University of Jerusalem, Rehovot 7610000, Israel

**Keywords:** biosensors, directed evolution, herbicide, flow cytometry, functional selection, PYL/RCAR

## Abstract

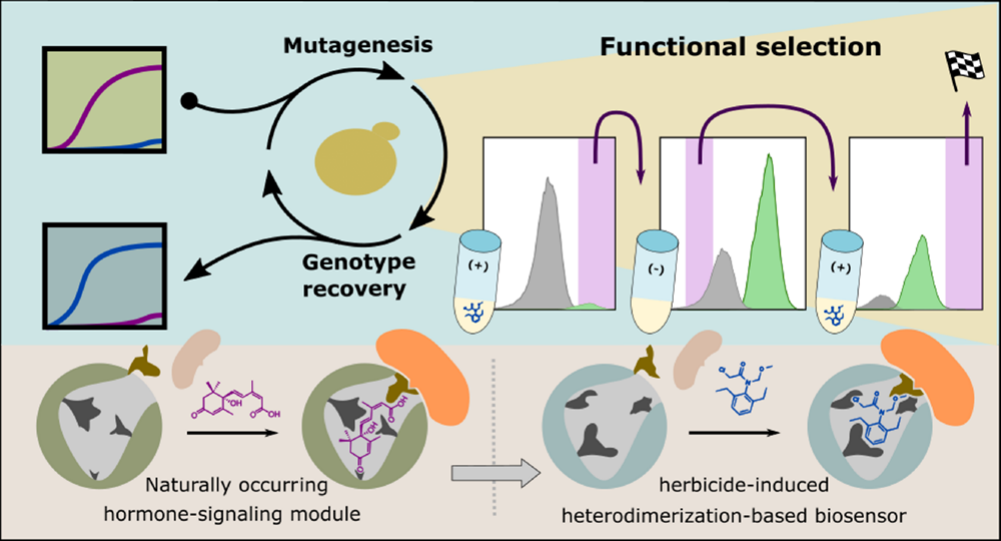

Developing sensory modules for specific molecules of interest represents a fundamental challenge in synthetic biology and its applications. A somewhat generalizable approach for this challenge is demonstrated here by evolving a naturally occurring chemically induced heterodimer into a genetically encoded sensor for herbicides. The interaction between PYRABACTIN-RESISTANT-like receptors and type-2C protein phosphatases is induced by abscisic acid—a small-molecule hormone in plants. We considered abscisic acid receptors as a potential scaffold for the development of biosensors because of past successes in their engineering, a structurally defined ligand cavity and the availability of large-scale assays for their activation. A panel of 475 receptor variants, mutated at ligand-proximal residues, was screened for activation by 37 herbicides from several classes. Twelve compounds activated at least one member of the mutant panel. To facilitate the subsequent improvement of herbicide receptors through directed evolution, we engineered a yeast two-hybrid platform optimized for sequential positive and negative selection using fluorescence-activated cell sorting. By utilizing this system, we were able to isolate receptors with low nanomolar sensitivity and a broad dynamic range in sensing a ubiquitous group of chloroacetamide herbicides. Aside from its possible applicative value, this work lays down conceptual groundwork and provides infrastructure for the future development of biosensors through directed evolution.

## Introduction

Sensing and responding to environmental or intrinsic small molecules is quintessential to various applications of synthetic biological systems. This requirement is typically fulfilled by the integration of a biological element that signals the presence of the target chemical.^1^ Biosensors enable a wide range of functions. Some obvious examples are the monitoring of molecules of interest in environmental, medical, or food samples, as a cheap and fast alternative to analytical methods.^2,3^ In microbial systems, genetically encoded small-molecule sensors may serve other, slightly more elaborate purposes. For example, metabolic fluxes in cell factories can be dynamically balanced by relaying the levels of intermediates. Alternatively, biosensors can support the directed evolution of proteins that affect the cellular concentration of their target chemicals (e.g., transporters and enzymes).^4^ With various possible applications, the prospect of tailoring a cellular sensor for a specific target molecule seems appealing in and of itself. However, with no single all-encompassing generic route, the requirement for an *ad hoc* methodology often complicates this beyond expediency.

In principle, this challenge can be met by modifying naturally occurring, chemically induced heterodimers (CIDs), to be inducible by a molecule of interest. The general aim of this work is to further demonstrate and explore how signaling proteins of the small-molecule hormone abscisic acid (ABA) may serve as viable candidates for this type of scheme. In plant cells, ABA elicits its effect by binding to a family of soluble receptors named PYRABACTIN RESISTANCE 1-LIKE/REGULATORY COMPONENTS OF ABA RECEPTOR (PYLs/RCARs), thereby stabilizing their closed-gate conformation.^5,6^ ABA-bound PYLs interact with type-2C protein phosphatases (PP2Cs), which, in turn, are inhibited, leading to a downstream signaling cascade. Because ABA is perceived by inducing an interaction between two soluble proteins, researchers often employ yeast two-hybrid (Y2H) assays to characterize ABA signaling components. In the work of Park et al.,^7^ semirandom mutagenesis and Y2H screens were used to evolve an ABA receptor to bind and subsequently be activated by a synthetic agrochemical. This ligand–receptor pair was used as an orthogonal activation pathway for ABA responses in transgenic plants. Here, we approach a similar challenge (i.e., modulating the ligand specificity of a PYL protein) but from a rather molecule-centric point of view, focusing on the development of biosensors for herbicides.

Herbicides are a class of agrochemicals used to control competing weeds in crops. Modern agriculture relies heavily on chemical weed control for retaining high productivity.^8^ This dependency poses several challenges, first among them is the persistence of residual levels of herbicides in the environment or in agricultural products, where they may cause undesirable secondary effects.^9^ The need to avoid damage to the crop plant while treating adjacent weeds represents an additional constraint.^10^ Traditionally, this problem is circumvented by exploiting the selective action of certain herbicides. A more modern solution entails the development of resistant crops, for example, bearing herbicide-detoxifying enzymes.^11,12^ Because herbicide-detoxifying enzymes may themselves be improved via directed evolution, we project that the ability to sense herbicides and relay their dynamic levels is of interest from both environmental and biotechnological perspectives.

The first phase of this work focused on identifying potential receptor scaffolds, which may serve as reporters for herbicides. In an initial screen, we tested 475 PYL mutants, each with a panel of 37 herbicides from several distinct classes. This led to the identification of ligand-activated receptors for 12 different herbicides. Each of these variants may conceivably be used as a stepping stone for future optimization to report their respective agrochemical agonist. In the second phase, we tackled a common impediment to the development of biosensors through directed evolution. Mutations that result in high basal (ligand independent) activity occur sporadically and are hard to predict, with PYL receptors specifically and for signaling proteins in general.^13^ The selective process is often complicated by a high rate of constitutively active variants, as they mask improved ligand-dependent variants. Additionally, even moderate basal activity is considered a negative attribute for biosensors, as it limits their signal amplitude and narrows their dynamic range. Therefore, to facilitate the subsequent optimization of herbicide receptors, we modified a Y2H platform, rendering it compatible with sequential positive and negative selections by fluorescence-activated cell-sorting (FACS). Utilizing this new platform, we managed to isolate sensitive and specific reporters for a heavily used and robust group of chloroacetamide herbicides. Isolated lines of yeast were later used as live sensors and successfully detected alachlor—a notorious environmental contaminant—mixed into complex soil samples at a dosage as low as 10 ppb, with minimal sample preparation and handling.

## Results and Discussion

### BdPYL3 and Several Single-Substitution Variants Are Activated by Herbicides from Distinct Chemical and Functional Classes

**Figure 1:**
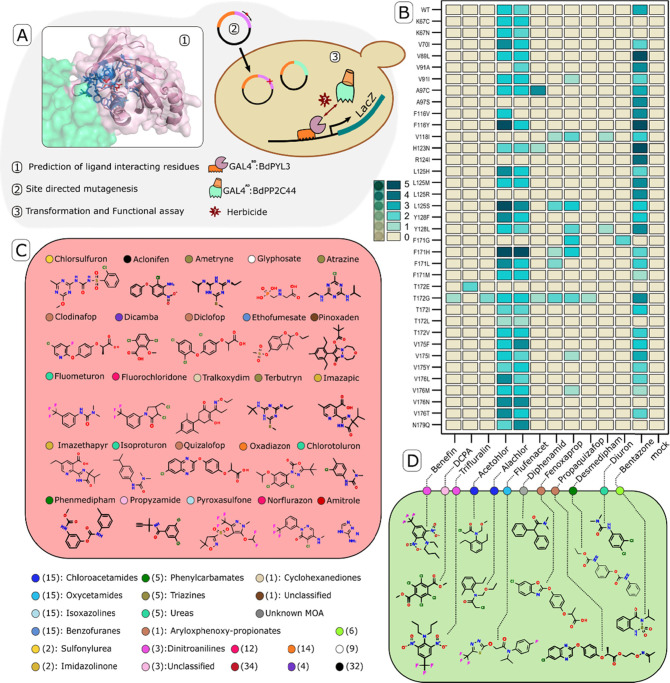
Chemical yeast two-hybrid screen of a BdPYL3-mutant panel. (A) Solved structure of a receptor/ABA/PP2C complex (AtPYL2, ABA, and AtHAB1 in pink, red, and green, respectively) was used to predict 25 ligand cavity lining residues (<5 Å from ABA, highlighted in blue).^15^ A collection of 475 pBD-GAL4 plasmids carrying all possible substitutions at these positions was created by site-targeted mutagenesis and transformed separately into a pACT2-BdPP2C44-harboring Y2H strain (Y190). All lines were challenged with each herbicide and tested for induced PYL-PP2C interaction by LacZ activity assay. Plates were photographed under consistent illumination, and staining intensity was assessed manually according to a fixed 0–5 intensity ladder. (B) A partial report of this screen, containing all ″hits″ is provided in a heat-map format, with each row specifying a variant and each column representing a different herbicide. Chemical structures of compounds, for which induced receptor variants were or were not identified, are shown in green or red rectangles, respectively (C, D). Color-coded circles assign herbicides to different groups according to HRAC classification, with mode-of-action (MOA) classes indicated in parentheses. Chemical families within MOA classes are also specified if more than one family was represented in the screen. Class 1 compounds (acetyl-CoA carboxylase inhibitors) were tested in their pro-herbicide form—see Table S1 for more details.

A screen was designed to identify PYL variants that recognize herbicides as agonists and can serve as possible leads for biosensor development. As a scaffold of choice, we selected a receptor/co-receptor pair from the model grass *Brachypodium distachyon* (*BdPYL3* and *BdPP2C44*, respectively), which was previously cloned, characterized, and confirmed to interact in an ABA dose-dependent manner.^14^ By homology, BdPYL3 groups with monomeric receptors, which generally display detectable basal activity in a Y2H-LacZ assay. We reasoned that basal BdPYL3-BdPP2C44 affinity, which is slightly below the detection threshold, coupled with the relative sensitivity of the LacZ assay, might enable the identification of weak agonists. Twenty-five ligand-interacting residues were predicted based on a solved structure of a receptor-ABA-phosphatase complex^15^ and subjected to saturation mutagenesis, aiming to expand the potential for alternative ligand recognition. All possible single amino acid substitutions at these positions were introduced by site-directed mutagenesis to a plasmid bearing BdPYL3 in fusion with a GAL4-DNA binding domain (GAL4^BD^). The 475 resulting plasmids were later individually transformed to a Y2H line expressing BdPP2C44 fused to the GAL4-activating domain (GAL4^AD^). We then screened the collection for induced BdPYL3-BdPP2C44 interaction in the presence of 37 herbicides from several functional and chemical groups (16,625 unique combinations). Receptor activation was tested by LacZ activity assays on solid media, each supplemented with one herbicide at a concentration of 100 μM (Figure 1A).

Three chemicals—bentazon, alachlor, and acetochlor—were found to act as weak agonists of the wild-type BdPYL3. The former is a class 6 photosystem II-inhibiting, thiadiazine herbicide. Alachlor and acetochlor are similar chloroacetanilide compounds, which share a mode of herbicidal action, that is, inhibition of very-long-chain fatty acid synthase (VLCFAS).^16^ In vitro assays of phosphatase inhibition by BdPYL3 confirmed that alachlor, acetochlor, and bentazon are true BdPYL3 agonists and do not operate indirectly or following some chemical modification in vivo (Figure S1). We tested three additional chloroacetamides, butachlor, pretilachlor, and S-metolachlor, each of which activated at least one receptor variant but not the wild-type receptor. The constructed collection of mutants expanded the range of detectable compounds by nine additional herbicides: one additional inhibitor of VLCFAS (from a different chemical subclass), two class-5 photosystem II inhibitors, two blockers of lipid synthesis via acetyl-CoA carboxylase, three microtubule assembly inhibitors, and one compound of unknown mode of action (Figure 1B, D). This high rate of hits (12/37) was somewhat surprising, considering the limited size of the mutant collection and the brute force approach we took in its design. However, given these results, coupled with the work of Park et al.^7^ and with the more recent study which utilized a homologous PYL/PP2C to monitor cannabinoids and organophosphates,^17^ naturally occurring ABA-sensory modules emerge as promising scaffolds for biosensor development.

Chloroacetamides are an extensively used class of pre-emergence herbicides. Their selective action is enabled in a limited range of crops by the activity of detoxifying enzymes. From an environmental perspective, chloroacetamides are suspected carcinogenic and demonstrated to negatively affect aquatic life upon leaching to natural reservoirs.^18^ Therefore, in the second phase of this research, we performed directed evolution of BdPYL3 to develop sensitive and specific receptors for chloroacetamides, which may have both biotechnological- and environmental health-related applications.

Follow-up screens were performed to identify synergistically acting combinations of mutations. Thirteen substitutions, which enhanced the BdPYL3 response to alachlor, acetochlor, or S-metolachlor, were randomly combined. Our shuffling methodology included the amplification of partial BdPYL3 fragments using mutagenic primers and re-assembly into a GAL4^BD^ plasmid using the Gibson isothermal reaction^19^ (Table S2). The resulting library was transformed into a GAL4^AD^:BdPP2C44-expressing Y2H strain bearing *envyGFP* as an additional fluorescent reporter for GAL4 reconstitution. This combinatorial library was screened manually by sampling fluorescent colonies on plates supplemented previously subactivating levels of alachlor, acetochlor, or S-metolachlor. In a subsequent rough screen, sampled clones were grown in 96-well plates, with or without their respective ligands. Notably, 897/1366 clones had basal fluorescence exceeding that of BdPYL3^WT^ by a factor of two. A follow-up screen based on the more sensitive LacZ staining assay was performed on 128 ligand-dependent fluorescent clones, 34 of which had detectable basal activity. Several improved chloroacetamide-responsive BdPYL3 variants were isolated for further optimization (Figure S2). In addition to their high rate within this library, constructed by shuffling nonconstitutively-activating single mutations, high-basal-activity variants were also common in the initial screen of single substitutions (18/475). The identities of these activating mutations were consistent with those reported in the past for a PYL homologue from *Arabidopsis thaliana*, which were demonstrated to stabilize the apo-receptor in an active-like closed-gate conformation.^13^ Taken together, the results suggested a high rate of constitutive mutants.

### A Modified Y2H Platform for Functional Selection by FACS

The prevalence of high-basal-activity variants posed a challenge for upscaling the directed evolution of BdPYL3. To effectively select for improved ligand-dependent variants, each round of evolution must include an additional negative selection step. We reasoned that effective reverse selection could be used both to eliminate high-basal-activity variants and to select against activation by chemically similar nontargets or for more elaborate conditionally activated variants. Some existing yeast platforms facilitate negative selection against protein–protein interactions in yeast. For example, the URA3 metabolic marker allows for both positive and negative selections, but for the purpose of biosensor engineering, its selectivity stringency is rigid and can only be arbitrarily predetermined.^20^ Another more modern system leverages deep sequencing to retroactively identify variants following positive metabolic selection.^21^ We decided to utilize the fluorescent Y2H-strain (MaV99 + GAL1/UAS_g_:envyGFP:ADH1_ter_@HO) and the more dynamic platform of FACS as a selection method. Several studies utilized FACS to improve the specificity and dynamic range of biosensors.^22,23^ Our rationale was that by using FACS, population-scale response to selective conditions can be assessed at each step and selectivity stringency can be adjusted ″on the fly″, by choosing an optimal combination of ligand concentrations and fluorescence intensity thresholds.

**Figure 2:**
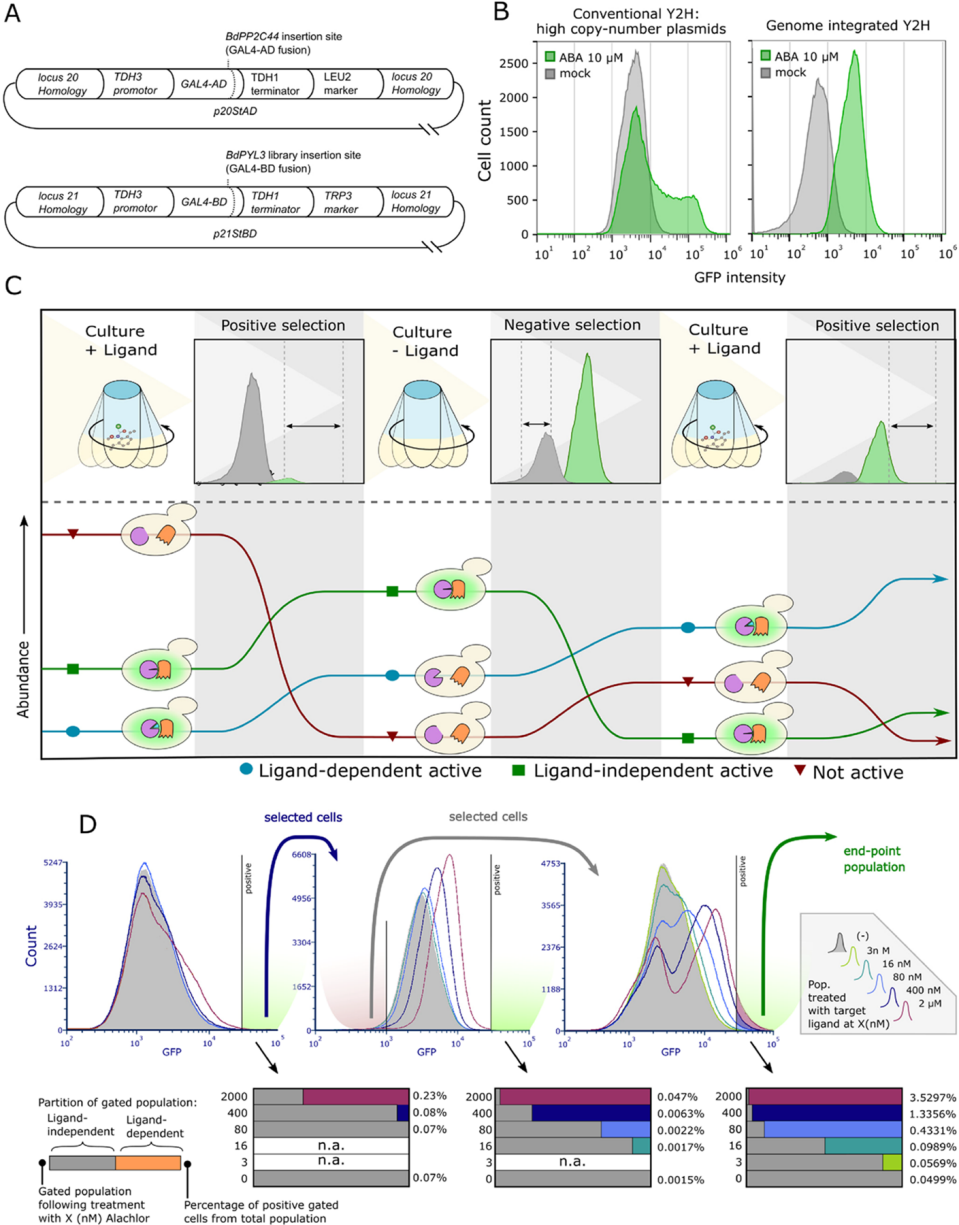
A FACS-based methodology for receptor engineering and its application for isolation of improved alachlor-responsive BdPYL3 variants. (A) Maps of two vectors for genome-integrated Y2H—p20StAD and p21StBD. (B) Population makeup of plasmid-encoded (left panel) vs genome-integrated Y2H (right panel). Gray and green histograms represent mock (DMSO-solvent) and ABA-treated cultures, respectively. (C) Outline of a conceptual protocol designed to enrich a rare population of improved ligand-responsive receptors out of complex libraries. Upper part describes three consecutive steps which comprise a single round of evolution. Lower part illustrates the dynamics of clones expressing three receptor architypes, along this process: improved ligand-responsive (cyan; circles), constitutively active (green; squares) and variants that are not activated within the given experimental context, that is, nonfunctional or inert receptors (red; triangles). (D) FACS-recorded data of population-scale dynamics during an exemplary round of selection. An error-prone library (average of 4 substitution/gene) was selected for improved ligand-dependent receptors for alachlor. Within each step, the same population was sectioned and treated with different doses of the target ligand (each represented by a histogram of ∼5 × 10^5^ events). The specific GFP intensity gate shown was not used de facto for sorting (in step II, for example, a negative gate was used to collect cells). Rather, it serves here as an arbitrary (yet consistent) threshold by which cells are retroactively categorized as positive or negative. The purpose of this experiment was to test how with each selective step clones expressing improved receptor variants are amplified and make up an increasingly larger fraction of the final population. Each horizontal bar represents the inferred partition of constitutive-positive vs ligand-induced positive clones, gated post-treatment with a given ligand concentration (specified on the left). The size of each gated population, expressed as a percentage of the total population, is indicated on the right side.

Preliminary assays highlighted a fundamental problem in applying Y2H at single-cell resolution. In the standard Y2H assay, interactors are expressed from two separate 2 μ plasmids that are subjected to stochastic copy number variance,^24^ meaning that ″weaker″ receptors may exert a stronger signal due to the amplification of receptor/phosphatase expression or vice versa. Additionally, these vectors may be lost, even while selective pressure is maintained, when cells are grown in batch (i.e., liquid culture or within colony).^25^ Thus, for a given clonal population, even under a positive condition (e.g., expressing a constitutively active variant), a large fraction of the cells is falsely ″OFF″ (Figure 2B, also Chen et al.^26^). These issues were expected to introduce an additional random factor affecting the signal intensity of any given cell and also to render negative selection unreliable. To circumvent this problem, we created an alternative set of integration plasmids for a genome-integrated Y2H system (Figure 2A). In these plasmids, the origin of replication is lacking and all yeast expression cassettes (i.e., GAL4 domains and auxotrophy markers) are flanked by homology sequences for guided homologous recombination. Target loci ″20″ and ″21″ are named according to a previous study that characterized them as facilitating high expression relative to a panel of similar integration sites.^27^ We also replaced the original promoter and terminator (from the *ADH1* gene) with the *TDH3* promoter and *TDH1* terminator, respectively, which together maximize expression in yeast.^24^ These adjustments were made to partially compensate for the decreased expression intensity naturally associated with a reduction in the gene copy number. FACS analysis was performed to compare between the genome-integrated Y2H system described above and a parallel plasmid-encoded standard Y2H line (both expressing GAL4^BD^:BdPYL3^WT^ and GAL4^AD^:BdPP2C44). These two homogenous populations were treated with or without ABA to simulate a receptor in ″ON″ and ″OFF″ states, respectively. As seen in Figure 2B, unlike the standard plasmid-encoded version, the alternative genome-integrated system was characterized by an overall reduced, yet highly uniform and stable signal.

Figure 2C outlines a conceptual process which we applied to enrich improved ligand-responsive variants after each round of mutagenesis. In Step I, the major bottleneck of the selective process, the top 0.01–0.05% of cells (in terms of GFP intensity) are collected from a liquid culture supplemented with previously subactivating levels of the target ligand. In our experience and with this stringency of selection, 10^3^–10^4^ out of 10^7^–10^8^ cells can be selected within a practical net runtime of 1–2 h. The resulting population is enriched for clones that express either constitutively active or improved ligand-responsive variants. In Step II, GFP-negative cells are sorted following growth in the absence of the target ligand (or under specific negative conditions). This step removes constitutively active clones and, by applying a lower threshold for sorting, can be used to select for variants with low basal activity. In step III, the population is pregrown in several liquid cultures spiked with a series of decreasing ligand concentrations. This, in turn, enables identification of the optimal combination of ligand concentration and fluorescence intensity threshold with which the top-performing variants can be enriched most effectively. Given its reduced complexity following two stringent positive selections, the end-point population can be screened on a clone-by-clone basis, using a more robust assay.

The added value of this selective process is exemplified by the experimental population-scale dynamics data recorded during the screening of high-complexity libraries (Figures 2D and S3). In the first case, an error-prone library was screened for alachlor-induced variants (Figure 2D). Retroactive analysis showed that with the given threshold, pretreatment with 80 nM alachlor did not increase the fraction of positive-gated cells, relative to the mock treatment, meaning that of the ∼5 × 10^5^ sampled events from the full library, 80 nM-induced cells were undetected. After the first positive selection, pretreatment with 16 nM alachlor and sorting with the same gate was projected to result in 12% 16 nM-responsive clones. Additional negative selection (following growth without alachlor) brought about a substantial benefit. With this twice-sorted population, ligand-dependent positives are inferred to account for 50 or 7% of the gated population, given that the population was pretreated with 16 or 3 nM alachlor, respectively. Figure S3 describes a screening of the same library, this time for improved S-Metolachlor reporters. Overall, based on these data, it seems that the BdPYL3 scaffold is less compatible with S-metolachlor as an agonist, as it took higher concentrations to detectably ″shift″ the original full-scale library. Additionally, positive gating after treatment with 0.4, 2, or 10 μM of S-metolachlor is expected to result in similar constitutive/induced ratios, whether performed on the original library or on a positively selected population. However, the exclusion of ligand-independent positive clones by an additional counter-selection is projected to have enabled sorting of a population composed of 20% clones responsive to 0.4 μM S-metolachlor. Taken together, these results demonstrate the relative strength of this platform in comparison to other methods that support both positive and negative functional selection. As a FACS-based assay, it is compact and generates single-cell-resolution data, which can inform the fine-tuning of target-chemical dosage and signal intensity thresholds in real-time. This in turn, maximizes the likelihood of retrieving the highest potential functionality (i.e., broadest dynamic range and signal amplitude) from each variant library.

### Directed Evolution of Receptors for Chloroacetamide Herbicides

As a steppingstone for further improvement, we chose a subset of nine variants that were isolated in a previous manual screen. These specific variants contained relatively a few fixed mutations in the central stretch of a sequence spanning seven ligand-proximal positions, for which several beneficial mutations were identified in the first screen (F116, V118, H123, R124, L125, Y128, and S130). Our approach for sequence diversification was semirandom, saturation mutagenesis at putative pocket lining residues and modular assembly by Gibson isothermal reactions. A pool of synthetic dsDNA fragments, corresponding to this segment and containing all possible double-substituted variants, was synthesized and reassembled with upstream and downstream fragments amplified from a pool of the nine background variants. The library of 10^5^-theoretical complexity was subjected to FACS selection, as described in Figure 2C, with initial positive selection following exposure to 400 nM alachlor. Several BdPYL3 variants isolated in this phase were responsive to different chloroacetamides at 10^2^–10^3^ nM but also tended to display increased basal activity (see Figures S2, S3 and Table S2). This could be explained by the fact that in the second step of this round of FACS, we used a rather promiscuous gate for negative selection (collecting the portion with the lower 20% of GFP intensity). We tested several of these variants in an independent in vitro phosphatase inhibition assay, which generally mirrored the patterns of ligand selectivity and sensitivity observed in the in vivo experiments (Figure S1).

**Figure 3:**
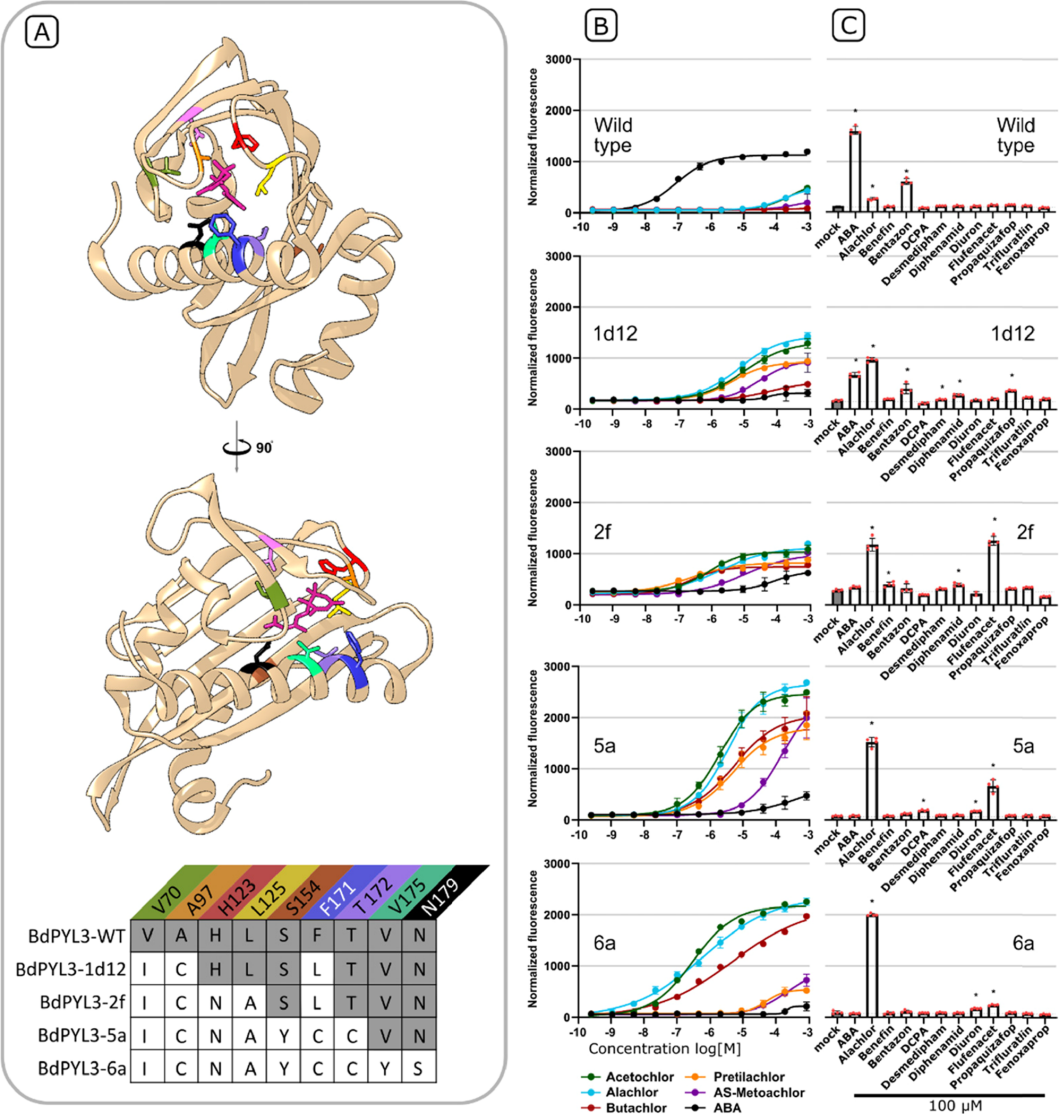
Improved chloroacetamide response and cross-reactivity of four variants isolated in consecutive rounds of mutagenesis and selection. (A) Two snapshots of a homologous ABA-bound PYL extracted from a crystal struture (PDB code 3KB3).^15^ ABA is highlighted in pink. Other colored stick models indicate residues that were mutated in one or more of the said variants. The table below details the substitutions present in each BdPYL3 variant. Color scheme for mutated positions is consistent with the structural snapshots above. (B, C) Plasmid-encoded Y2H assay with GAL4-regulated mScarlet-I as a reporter gene in a 96-well plate format. (B) Dose response of each variant to ABA and five chloroacetamides. (C) Nonspecific activities were tested by treating each variant with a panel of herbicides at 100 μM (DMSO served as mock). All 12 herbicides activated at least one BdPYL3 mutant in the initial screen. The elevated basal activity of variants 1d12 and 2f is evident by their fluorescence in the mock treatment. Asterisks indicate a statistically significant difference from the mock treatment (*p*-value < 0.05, Dunnett’s test). In vivo binding curves of other isolated variants and of an isogenic line bearing an empty pBD-GAL4 vector are provided in Figure S4. The *Y* axes are uniform and shared across all plots in panels B and C.

Our next aim was to improve these isolated variants by achieving a lower threshold of detection, while tackling the aforementioned issue of basal activity. A similar sequence diversification strategy was taken in creating libraries 7 and 8 (Table S2), which were pooled and screened by a similar scheme of cell sorting. This time, positive selection was achieved following exposure to 160 nM alachlor or acetochlor, and subsequent negative selection was rendered more stringent to select for variants with an especially low basal signal (collecting the lower 2% of cells). The thrice-sorted population was dominated by clones expressing two variants—BdPYL^5a^ and BdPYL3,^5b^ which did not display a substantially lower detection threshold, but had an improved signal amplitude, that is, a low basal signal and a high signal at saturation (Figure 3). Both sequences were derived from BdPYL^2f^ and had double substitutions at residues L171 and T172. Variant 5a also contained a spontaneous S154Y substitution. Phosphatase inhibition in vitro assays were not performed from this point onward, as we were unsuccessful in purifying these receptors in an active state. To build upon BdPYL3-variants 5a and 5b, we constructed library 9 by randomizing residues V175, V176 and N179, which, like residues 171 and 172, project into the ligand cavity from an α-helix (Figure 3A). This library was screened, with initial positive selection performed after 50 nM alachlor treatment. BdPYL3 variant 6a, which was isolated in this screen, is derived from variant 5a, and characterized by an equally broad signal amplitude, dynamic range, and a detection threshold of 1–10 nM (Figure 3B). Overall, three rounds of evolution using the proposed selective scheme achieved a 100-fold improvement in BdPYL3 sensitivity for chloroacetamide and a reduction in the basal signal relative to the starting-point variant (BdPYL3^1d12^).

Our working hypothesis was that selecting for improved response to a specific target-ligand would bring about a reduced response to other substances. To assess this possibility, we tested four consecutively derived BdPYL3 variants for induced BdPP2C44 binding in the presence of 10 other herbicides that activated single mutants in the initial screen. Given that FACS selections were mainly based on response to alachlor, we expected to observe an overall trade-off between alachlor sensitivity and the acceptance other herbicides as agonists. However, the displayed response profiles only partially reflect this expectation. While activation by the tested nonchloroacetamide chemicals was relatively residual in the most-sensitive variant (BdPYL3^6A^), in some steps (variants BdPYL3^1d12^ and BdPYL3^2f^), increased response to alachlor seemed to accompany an overall rise in ligand promiscuity (Figure 3C). We suggest that future projects can include pretreatment with chemically similar nontarget compounds, prior to the negative selection in step II. This should render specificity an achievable goal in and of itself.

### Alachlor Measurement in a Complex Sample

**Figure 4:**
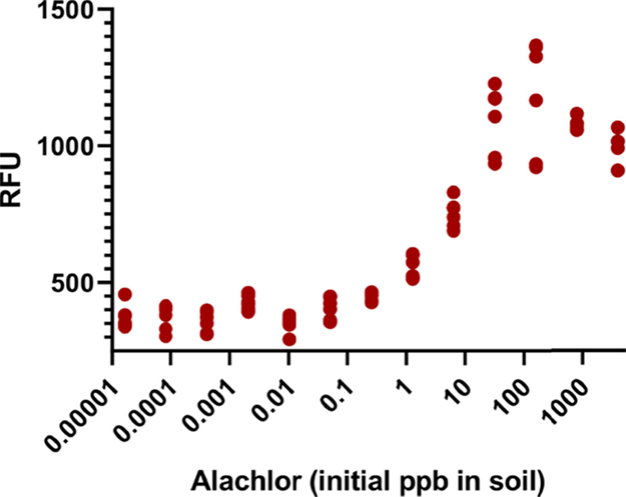
Five weeks after being mixed into a composite potting soil, 10 ppb alachlor was detectable using a highly simplified assay with minimal sample preparation. The assay utilized a Y2H stain harboring mScarlet-I as an additional reporter and plasmid-encoded copies of GAL4-BD:BdPYL3^6A^ and GAL4-AD:ΔNBdPP2C44. Small pots with Arabidopsis seedlings containing an equal mass of composite soil were drenched in ALANEX (480 g/L alachlor) diluted in tap water to appropriate levels. Pots were allowed to dry up and were watered occasionally for 5 weeks. Sample preparation is described in the experimental section. Each data point is an average of 4 replicates, which are the same yeast culture mixed with the same soil sample and sectioned to four different wells for growth.

The detection sensitivity of our reporter strain for alachlor was low in comparison to other proposed detection methods. For example, the study of Segal et al.^28^ reported on sub-ppb thresholds of detection for alachlor and S-metolachlor in aqueous solution using a tailored plasmonic optical sensor. However, using yeast lines as live sensors for herbicides in environmental or food samples offers certain potential advantages. First, it carries potential resilience to a matrix effect and fluctuating conditions, which may enable its use in complex samples. Second, microbial reporters may be applied using highly simplified protocols. To demonstrate the detection of alachlor in complex samples, 13 dilutions of a commercial alachlor formulation were mixed into pots containing a composite potting soil. Five weeks after mixing, 10 ppb alachlor was still significantly detectible using an extremely simplified protocol (Figure 4). Sample preparation for analysis included short mixing of soil into a liquid yeast medium, rough filtration, and 16 h of incubation before readout. No sample extraction or concentration were performed. A recent work that utilized a homologous PYL/PP2C module for biosensor development also demonstrated the detection of target analytes in complex samples (e.g., urine, blood, and saliva).^17^ Additionally, it was shown that this module is readily transferable to multiple methods of output generation, including enzyme-linked immunosorbent-like assay and luciferase fragment complementation.^17^ It is not far-fetched to assume that additional iterations of directed evolution as well as more elaborate protocols of sample preparation and readout will improve the sensitivity of this herbicide-detection method to applicably relevant levels.

## Conclusions

Streamlining the development of genetically encoded biosensors may benefit numerous areas of research, industry, and public and environmental health. The successful identification of chemically induced BdPYL3 scaffolds for 12/37 herbicides demonstrates their specific value, along with their PP2C binding partners, as raw materials for biosensor engineering. In many plants, PYL receptors are encoded by a large family of genes. From that perspective, the natural variation in plant genomes can be viewed as a treasure trove of potential scaffolds. Tapping into this potential is burdened by the specific tendency of PYL receptors to assume a ligand-independent active conformation. This is likely a consequence of them arising from a nonligand-regulated common ancestor.^29^ The methodology we developed tackles this problem by leveraging the advantages of two distinct platforms. Yeast two-hybrid is an accessible, decades-established, and highly scalable functional assay. FACS supports both positive and negative miniaturized selections, while enabling informed real-time adjustments of selective conditions. The detection of alachlor in complex soil samples has applicative scientific value because it serves as a preliminary proof of concept for the application of chemically induced yeast two-hybrid lines for the detection of environmental contaminants.

## Experimental Section

### DNA Sequences, Cloning, and Mutagenesis

pBD-GAL4 and pACT plasmids (Clontech, CA, USA) bearing codon-optimized sequences of *BdPYL3* and ΔN*BdPP2C44* (encoding amino acids 121–480) were prepared in a previous work.^14^ Amplification of all DNA fragments was performed using Phusion High-Fidelity DNA polymerase (New England Biolabs, MA, USA). See supplementary Tables S2, S4, and S5 for more details on DNA sequences, plasmids, and primers used in this work. Unless specified otherwise, all multifragment assemblies and cloning procedures were performed using Gibson isothermal assembly reaction.^19^ All restriction enzymes used for plasmid linearization were purchased from New England Biolabs (MA, USA). Site-directed mutagenesis and mutagenesis by error-prone were performed using QuikChange Lightning Multi Site-Directed and the GeneMorphII reaction kits (Agilent, CA, USA), respectively.

### Yeast Transformation, Genome Editing, and Two-Hybrid Assays

Strains derived from MaV99 and Y190 were maintained on synthetic minimal (SD) media supplemented with Brent and CSM amino acid mixtures. Rich YPD medium was used to isolate or periodically maintain G418 resistance markers. Yeast transformations were executed according to standard or high-efficiency PEG/LiAC/carrier-DNA prtotocols.^30^

A background strain for FACS assays was engineered by integrating the following two constructs into the genome of strain MaV99. The coding sequence of envyGFP was assembled between the GAL1/UAS_g_ promoter and ADH1 terminator sequences into the pHO integrating vector.^31^ The sequence of ΔN*BdPP2C44* was cloned into p20StAD (Figure 2A). Resulting plasmids were linearized and transformed, and target site integration was verified by PCR with flanking and internal primers. For plasmid-encoded, fluorescence output Y2H assays, a GAL4-regulated mScarlet-I was added to the Y190 strain (constructed as described above for *envyGFP*-bearing MaV99).

Two-hybrid assays were performed either by LacZ activity staining, as described,^14^ or in a 96-well plate format for fluorescence intensity readout. To measure fluorescence intensity, liquid cultures (initial OD_600_ = 0.05) of a given strain were supplemented with each chemical treatment and placed in a Titramax shaker (Heidolph, DE) for 24 h, at 1100 rpm, 30 °C. envyGFP and mScarlet-I fluorescence were measured using a Synergy-H1 microplate reader (Biotek, VT, US) at excitation/emission wavelengths of 480/525 and 570/605 nm, respectively.

### Fluorescence-Activated Cell Sorting

Libraries of p21StBD bearing BdPYL3 variants were linearized (*ScaI* and *NgoMIV*) and transformed into the aforementioned FACS-compatible Y2H strain. Colonies were grown for three days on selective minimal agar, and library sizes were estimated. Colonies were then washed from the plates and grown in liquid SD (-Leu-Trp) supplemented with test chemical reagents. Prior to sorting, cells were harvested by centrifugation and resuspended in ice-cold PBS buffer to arrest growth. Data recording and cell sorting were performed using a BD FACSMelody (BD Bioscience, San Jose, CA) in purity modes at ∼7000 events per second. The target count was established to cover at least X10 the deduced complexity of each population. Gates based on forward- and side-scatter (FSC and SSC) were used to exclude doublets and to focus on a uniform population, thereby reducing cell cycle- and cell size-related signal intensity variance (FSC-A/SSC-A, SSC-H/SSC-W, and FSC-H/FSC-W in tandem). Sorted cells were either regrown in liquid for subsequent FACS or plated on dropout plates for clone recovery. Smaller scale verification screens were performed by LacZ staining and/or fluorescence in 96-well plates.

### Soil Analysis

Various dilutions of ALANEX (ADAMA, IL) were mixed into composite arabidopsis growth mixture composed of ∼75% white peat, ∼25% perlite, and micronutrients (substrate code 686 from Klasmann-Delimann, Geeste, DE). Mixing into the soil was performed by drenching. Dilutions and volumes were calculated to cover a range of doses (per gram of soil) from field-recommended-down to sub-ppb levels. After 5 weeks and three rehydration cycles, we allowed the soil to dry under ambient conditions, before sampling. A proportional volume (10 mL/g) of synthetic yeast growth medium was mixed and incubated with the soil while shaking for 10 min. The mixture was then filtered through a gauze pad-stuffed 30 mL syringe and centrifuged at 3000 *g* for 10 min to allow small particles to settle. The liquid medium was dispensed into 96-well plates, which was subsequently inoculated with fresh culture of an alachlor reporter strain (Y190 + GAL1/UASg:mScarlet:ADH1_ter_@HO, pACT-BdPP2C44, pBD-BdPYL3^6A^) to initial OD_600_ of 0.05. We then allowed the cultures to grow for 16 h prior to readout.

## Supporting Information Available

Methods for protein purification and in vitro phosphatase inhibition assays; detailed list of tested herbicides (Table S1); list of amino-acid substitutions (Table S3) and supplementary tests of isolated chloroacetamide receptors, including phosphatase inhibition (Figure S1); LacZ staining (Figure S2) and *in vivo* binding curves (Figure S4); additional information of DNA parts and manipulation including an outline of library construction (Table S2), primers (Table S4), and plasmids (Table S5); and population-scale dynamics during selection for S-metolachlor-activated receptors (Figure S3) (PDF)
