# Supplementary material for: Directed Evolution of Herbicide Biosensors in a Fluorescence-Activated Cell-Sorting-Compatible Yeast Two-Hybrid Platform
Source: ACS Synth Biol. 2022 Aug 3;11(8):2880–8. doi: 10.1021/acssynbio.2c00297 (PMC9396700; doi:10.1021/acssynbio.2c00297)
Supplement: Supplementary file 1 — sb2c00297_si_001.pdf [file sb2c00297_si_001.pdf]

Supplementary information

Directed evolution of herbicide biosensors in a FACS-compatible yeast two-hybrid platform

Gil Zimran, Erez Feuer, Oded Pri-Tal, Michal Shpilman and Assaf Mosquna*

The Robert H. Smith Institute of Plant Sciences and Genetics in Agriculture, The Hebrew University of Jerusalem, Rehovot 7610000, Israel.

*Corresponding author

Supplementary methods

Phosphatase inhibition assay

The coding sequences of BdPYL3 and AtHAB1 were cloned into pET28 (EMD Biosciences), allowing the expression of a 6XHIS-tagged protein in transformed BL21 (DE3) *pLYS* (Promega, www.promega.com). For AtHAB1 purification, 1 L LB was inoculated with 50 ml overnight culture and grown at 30 °C, 200 rpm. At OD_{600}=0.4, $MnCl_2$ was added to a final concentration of 4 mM. At OD_{600}=0.9, the culture was briefly placed on ice to arrest bacterial growth, and supplemented with 1 mM IPTG to induce recombinant protein expression. Cultures were maintained overnight at 23°C/200 rpm, prior to cell harvesting and resuspension in 3 ml/g TBS [50 mM TRIS, 150 mM NaCl, pH 7.5] supplemented with 10 mM imidazole, 10 mM $MnCl_2$ and 1 tablet/50 ml of cOmpleteTM Protease Inhibitor Cocktail (Sigma). Cell suspensions were lysed by two rounds of freezing and thawing, sonicated and centrifuged. Lysates were subsequently passed through 3 ml Ni-NTA beads (QIAGEN) and washed by passing 60 volumes of suspension buffer with 30 mM imidazole. Phosphatase was eluted with suspension buffer containing 250 mM imiodazole and 1 mM tris(2-carboxyethyl)phosphine (TCEP). Overnight dialysis was performed in TBS buffer supplemented with 10 mM $MnCl_2$, 1 mM TCEP and glycerol 20%. Purification of BdPYL3 variants was performed similarly with the following differences: pre-growth and expression were performed in TB without $MnCl_2$. In addition, following IPTG induction, the cultures were maintained at 15°C/200 rpm. Further, rather than TBS, Buffer A [50 mM NaH2PO4, 300 mM NaCl, pH 8.0 and tablet/50 ml cOmpleteTM] was used for lysis, wash and elution steps (imidazole concentration and volume-ratios for each step were identical). Buffer was exchanged to TBS (without $MnCl_2$) by overnight dialysis.

The phosphatase inhibition reaction included 33 mM Tris acetate (pH 7.9), 66 mM potassium acetate, 0.1% bovine serum albumin, 10 mM MnCl2, 0.1% β-ME, 50 mM pNPP and increasing concentrations of either ABA or other tested ligands. To ensure consistency between preparations, phosphatase reactions contained a volume of purified 6XHIS-HAB1-GFP which resulted in ΔOD^{405} +0.5 in a 2-min receptor-free reaction. Reactions containing a saturating amount of ligand (100 μM) and increasing volumes of receptor were performed to experimentally establish an effective 1:1 PYL:phosphatase ratio for each purified batch of the BdPYL3 variants. Reaction mixture was allowed to equilibrate for 5 min prior to addition of substrate. Rate of pNPP hydrolysis by phosphatase was inferred from ΔOD^{405} during 5 min of the reaction at linear phase. Values represent percentage activity compared with phosphatase activity without a receptor.

Supplementary figures and tables

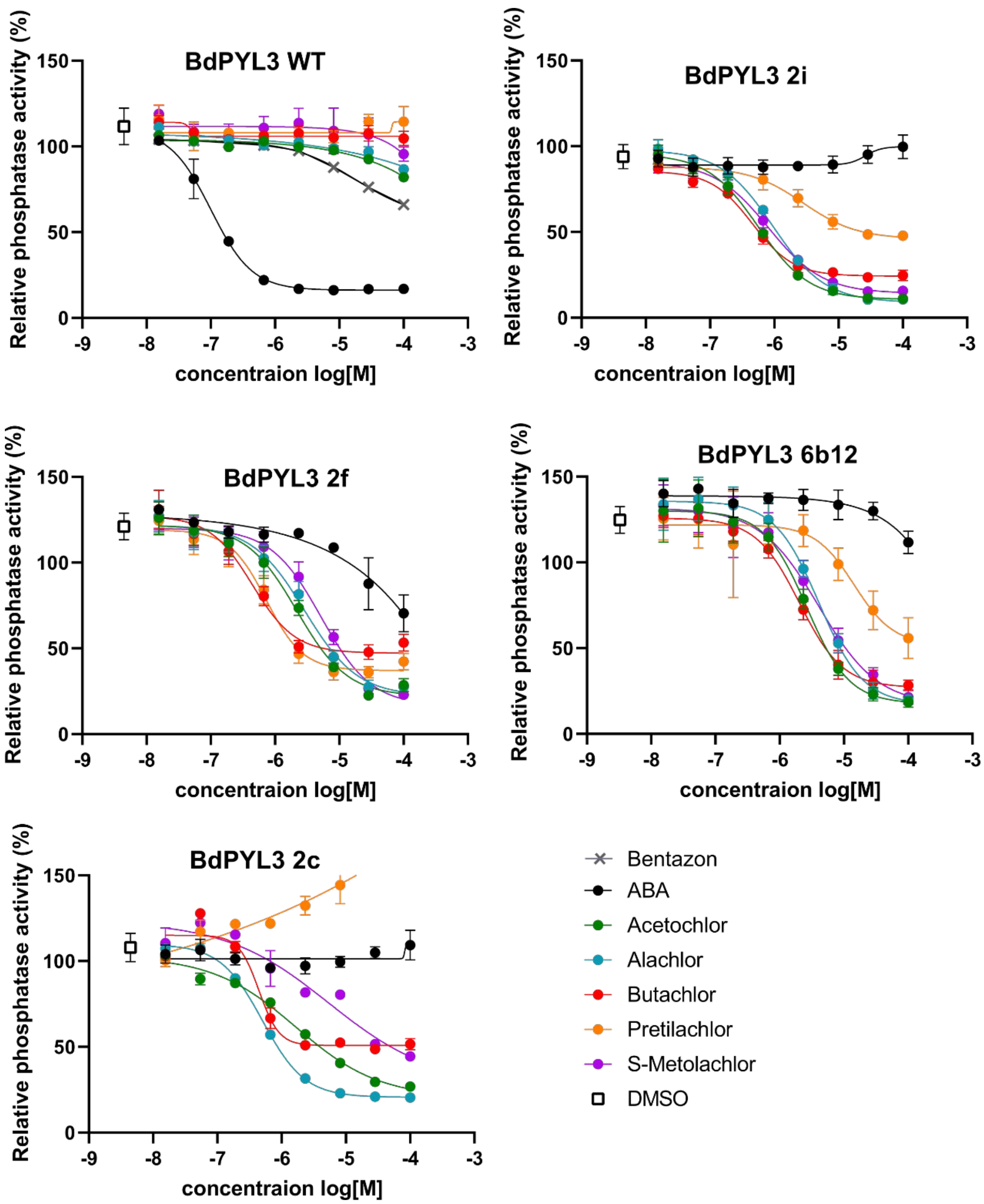

Figure S1 – Dose-dependent inhibition of recombinant 6XHIS-AtHAB1 (PP2C-type phosphatase) by recombinant 6XHIS-BdPYL3^{WT} and other recombinant variants. The effect of Alachlor, Acetochlor and Bentazon in this in vitro assay confirms that these herbicides act as true BdPYL3^{WT} agonists and do not opertate indirectly or following chemical modification in vivo. Findings regarding the improved response of several BdPYL3 variants to chloroacetamides are reflected in this indedpendent in vitro assay. An exception was the reverse response of BdPYL3^{2c} to Pretilachlor, which was reproduced in multiple repeated assays. AtHAB1 activity is expressed as percentage of a reaction in the absence of a receptor. Further information regarding this assay is provided in supplementary materials and methods.

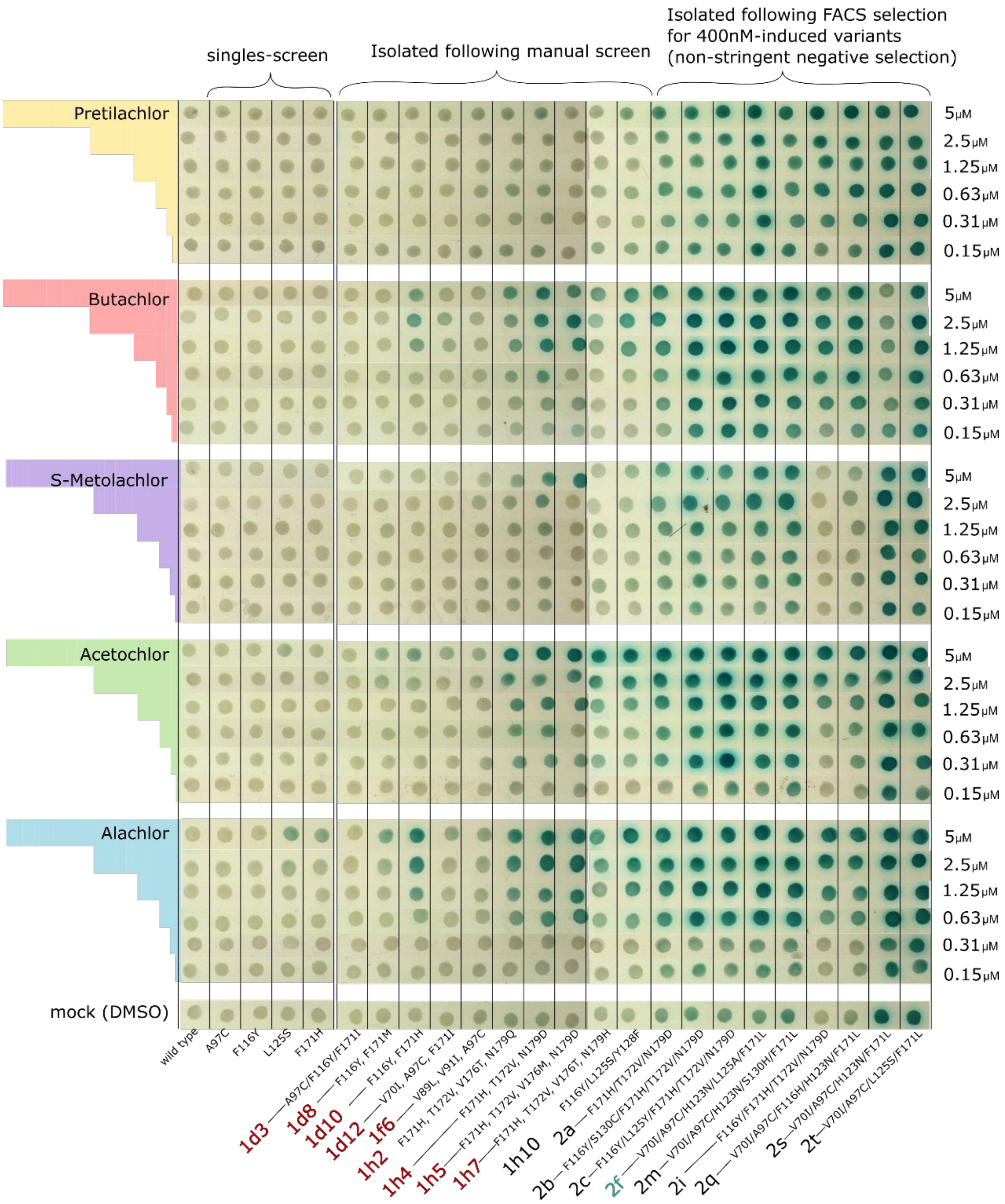

Figure S2- Summary of the Y2H assay of BdPYL3 variants isolated up to and including the first FACS-based selection performed in this work. Lines expressing different variants were dotted on synthetic dextrose medium suplemented with the specified chemical treatment, and allowed to grow for 2 days. Receptor-phsophatase interactions were assayed by measuring the expression of the LacZ reporter gene through LacZ/X-gal activity staining. Variations in the original BdPYL3WT polypeptide sequence are listed below each column. Identifiers for different variants are specified below if used in the main or supplemnetaty text. Variants which were the background for the construction of library #6 are indicated in red. Variants 5a and 5b were derived from variant 2f, which is highlighted here in cyan.

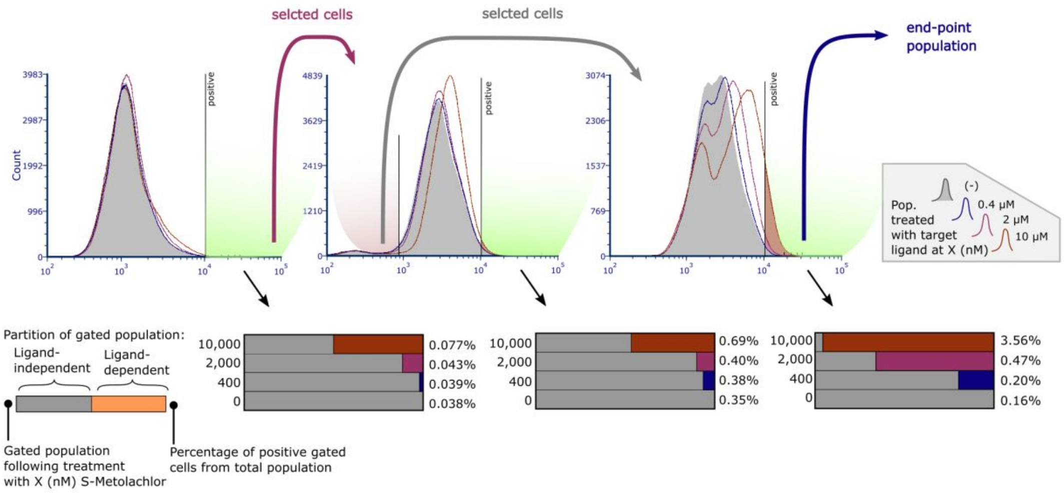

Figure S3 – FACS-recorded data of population-scale dynamics during an exemplary round of selection. An error-prone library (average of 4 substitutions/gene) was selected for improved ligand-dependent receptors for S-Metolachlor. Within each step, the same population was sectioned and treated with different doses of the target ligand (each represented by a histogram of ~5×10^5 events). The specific GFP intensity gate shown was not used de facto for sorting (in step II, for example, a negative gate was used to collect cells), rather, it served as an arbitrary (yet consistent) threshold by which cells were retroactively categorized as positive or negative. The purpose of this experiment was to test how with each selective step, clones expressing improved receptor variants, are amplified and make up an increasingly larger fraction of the final population. Each horizontal bar represents the inferred partition of constitutive-positive vs ligand-induced positive clones, gated post-treatment with a given ligand concentration (specified on the left). The size of each gated population, expressed as a percentage of the total population, is indicated on the right side.

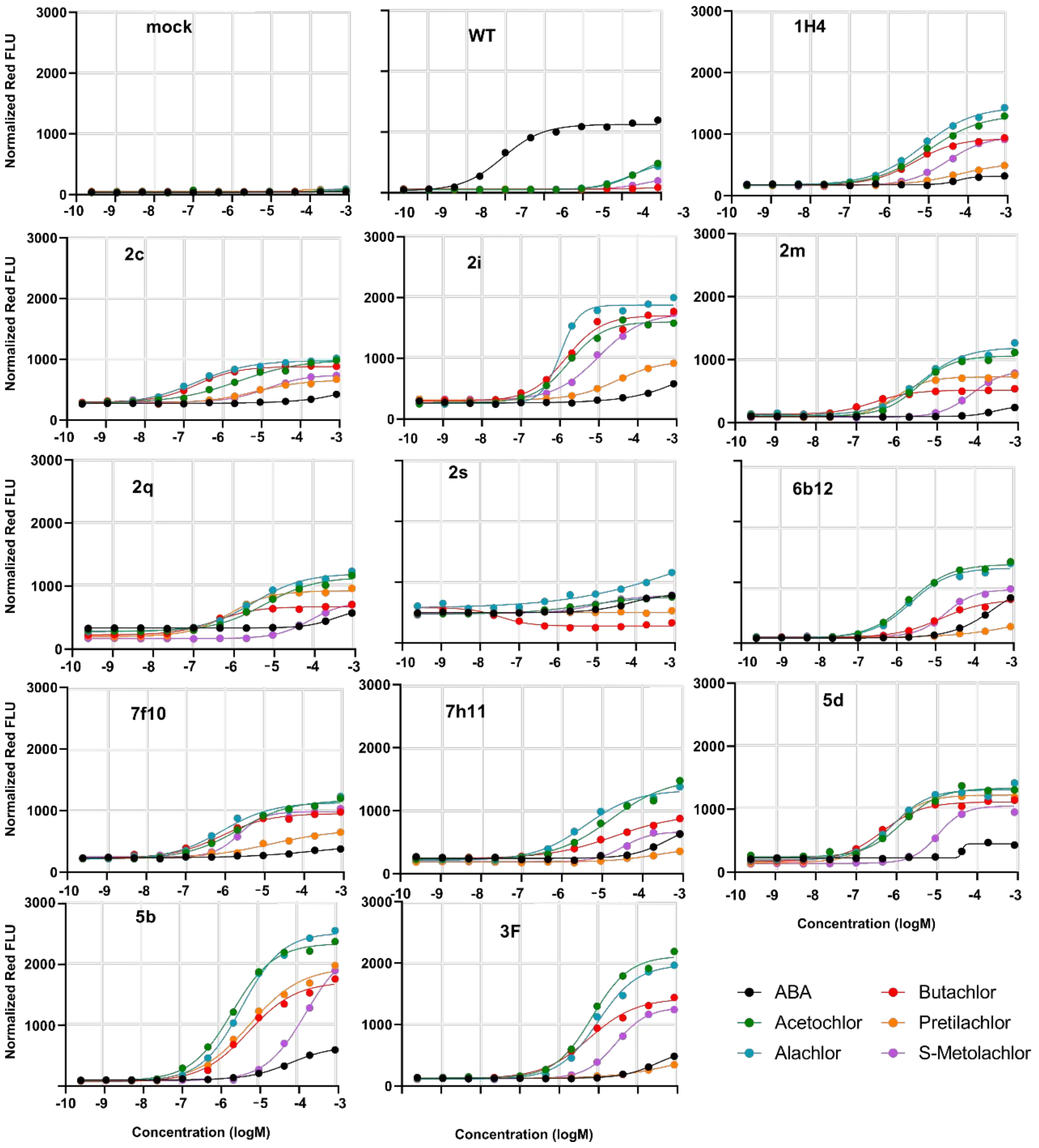

Figure S4 – In vivo binding curves of receptor variants not presented in the main text. Chloroacetamide-responsive variants were isolated during various stages of this study. Readout of receptor-phosphatse interactions is generated by a plasmid-encoded Y2H system with GAL4-regulated mScarlet-I as a reporter gene, in 96-well plates. The mock line was isogenic to the other yeast lines but harbored an empty pBD-GAL4 vector.

Table S1 – Herbicides tested in the single-substitution mutant screen and other experiments in this study[1].

Compound name	CAS identifier	HRAC classification	Mode of action	Chemical sub-class
Acetochlor	34256-82-1	15	Inhibition of Very Long-Chain Fatty Acid Synthesis	a-chloroacetamide
Aclonifen	74070-46-5	32	Inhibition of Solanesyl Diphosphate Synthase	
Alachlor	15972-60-8	15	Inhibition of Very Long-Chain Fatty Acid Synthesis	a-chloroacetamide
Ametryne	834-12-8	5	Inhibition of Photosynthesis at PS II D1 Serine 264 binders (and other non-histidine 215 binders)	Triazines
Amitrole	61-82-5	34	Inhibition of Lycopene Cyclase	
Atrazine	1912-24-9	5	Inhibition of Photosynthesis at PS II D1 Serine 264 binders (and other non-histidine 215 binders)	Triazines
Benefin	1861-40-1	3	Inhibition of Microtubule Assembly	Dinitroanilines
Bentazone	25057-89-0	6	D1 Histidine 2Inhibition of Photosynthesis at PS II<br>15 binders	
Butachlor	23184-66-9	15	Inhibition of Very Long-Chain Fatty Acid Synthesis	a-chloroacetamide
Chlorsulfuron	64902-72-3	2	Inhibition of Acetolactate Synthase	Sulfonylurea
Chlortoluron	15545-48-9	5	Inhibition of Photosynthesis at PS II D1 Serine 264 binders (and other non-histidine 215 binders)	Ureas
Clodinafop-Propargyl	105512-06-9	1	Inhibition of Acetyl CoA Carboxylase	Aryloxphenoxy-propionates
DCPA (Dacthal)	1861-32-1	3	Inhibition of Microtubule Assembly	
Desmedipham	13684-56-5	5	Inhibition of Photosynthesis at PS II D1 Serine 264 binders (and other non-histidine 215 binders)	Phenylcarbamates
Dicamba	1918-00-9	4	Auxin mimics	Benzoates
Diclofop-methyl	51338-27-3	1	Inhibition of Acetyl CoA Carboxylase	Aryloxphenoxy-propionates
Diphenamide	957-51-7	Ø	Unknown Mode of Action	
Diuron	330-54-1	5	Inhibition of Photosynthesis at PS II D1 Serine 264 binders (and other non-histidine 215 binders)	Ureas
Ethofumesate	26225-79-6	15	Inhibition of Very Long-Chain Fatty Acid Synthesis	Benzofuranes
Fenoxaprop-ethyl	66441-23-4	1	Inhibition of Acetyl CoA Carboxylase	Aryloxphenoxy-propionates

[1] Assignment of herbicides to mode-of-action classes and chemical sub-classes was according to Herbicide Resistance Action Committee (HRAC) classification[1].

Flufenacet	142459-58-3	15	Inhibition of Very Long-Chain Fatty Acid Synthesis	α-Oxyacetamides
Fluometuron	2164-17-2	5	Inhibition of Photosynthesis at PS II D1 Serine 264 binders (and other non-histidine 215 binders)	Ureas
Flurochloridone	61213-25-0	12	Inhibition of Phytoene Desaturase	N-Phenyl heterocycles
Glyphosate	1071-83-6	9	Inhibition of Enolpyruvyl Shikimate Phosphate Synthase	
Imazapic	104098-48-8	2	Inhibition of Acetolactate Synthase	Imidazolinone
Imazethapyr	81335-77-5	2	Inhibition of Acetolactate Synthase	Imidazolinone
Isoproturon	34123-59-6	5	Inhibition of Photosynthesis at PS II D1 Serine 264 binders (and other non-histidine 215 binders)	Ureas
Norflurazon	27314-13-2	12	Inhibition of Phytoene Desaturase	N-Phenyl heterocycles
Oxadiazon	19666-30-9	14	Inhibition of Protoporphyrinogen Oxidase	N-Phenyl-oxadiazolones
Phenmedipham	13684-63-4	5	Inhibition of Photosynthesis at PS II D1 Serine 264 binders (and other non-histidine 215 binders)	Phenylcarbamates
Pinoxaden	243973-20-8	1	Inhibition of Acetyl CoA Carboxylase	
Pretilachlor	51218-49-6	15	Inhibition of Very Long-Chain Fatty Acid Synthesis	a-chloroacetamide
Propaquizalofop	111479-05-1	1	Inhibition of Acetyl CoA Carboxylase	Aryloxphenoxy-propionates
Propyzamide (pronamide)	23950-58-5	3	Inhibition of Microtubule Assembly	Dinitroanilines
Pyroxasulfone	447399-55-5	15	Inhibition of Very Long-Chain Fatty Acid Synthesis	Isoxazolines
Quizalofop-p-ethyl	100646-51-3	1	Inhibition of Acetyl CoA Carboxylase	Aryloxphenoxy-propionates
S-Metolachlor	87392-12-9	15	Inhibition of Very Long-Chain Fatty Acid Synthesis	a-chloroacetamide
Terbutryn	886-50-0	5	Inhibition of Photosynthesis at PS II D1 Serine 264 binders (and other non-histidine 215 binders)	Triazines
Tralkoxydim	87820-88-0	1	Inhibition of Acetyl CoA Carboxylase	Cyclohexanediones
Trifluralin	1582-09-8	3	Inhibition of Microtubule Assembly	Dinitroanilines

Table 2 – Outline of library construction

#	Library	Fixed mutations/ background variants	Randomized residues	Fragments	PCR amplification: Primers FWD	PCR amplification: Primers REV	PCR amplification: Template	Theoretical complexity	screen	isolates
1	Singles around A97C shuffled	A97C	70-V/I, 89-V/L	1	250	67	A97C	101	Libraries pooled, plated on 5uM Alachlor, Acetochlor or S-Metolachlor.<br>Manual screen under a fluorescent stereoscope.	1F6
				2	68	251				
2	Singles around F116Y shuffled	F116Y	118-V/I, 123-H/N, 124-R/M, 128-Y/F/L,	1	250	69	F116Y	10 2		1H10
				2	70	251				
3	Singles around L125S shuffled	L125S	118-V/I, 123-H/N, 124-R/M, 128-Y/F/L	1	250	69	L125S	10 2		
				2	71	251				
4	Singles around F171H shuffled	F171H fixed	172-T/I/M/G/V/A/L/R/S, 175-V/F, 175-V/M/N/T, 179-N/D/Q	1	250	349	F171H	2.2*102		1D10<br>1H2<br>1H4<br>1H5<br>1H7
				2	72/73	251				
5	All singles shuffled	-	70-V/I, 89-V/L118-V/I, 123-H/N, 124-R/M, 128-Y/F/L118-V/I, 123-H/N, 124-R/M, 128-Y/F/L, 172-T/I/M/G/V/A/L/R/S, 175-V/F, 175-V/M/N/T, 179-N/D/Q	1	250	241	WT, K67C, K67N, V70I, pooled	1.5*103		1d3<br>1d8<br>1d10<br>1d12
				2	240	243	WT, V89L, V91I, A97C pooled			
				3	242	245	WT, F116Y, V118I, H123N, L125S/H/R, Y128F/L pooled			
				4	244	251	WT, V171H/I/L, T172 I/V/L/G, V175F, V176M, N179D pooled			
6	segment C randomized 1	1d3, 1d8, 1d10, 1d12, 1f6, 1h2, 1h4, 1h5	116, 118, 123, 124, 125, 128, 130 – all substitutions with custom synthesized dsDNA fragment pool*	1	250	243	1d3, 1d8, 1d10, 1d12, 1f6, 1h2, 1h4, 1h5, Pooled	7.8*105	3 FACS rounds:<br>Positive after 0.4uM ala, negative after dmso (lower 20%)	2c<br>2F<br>2I
				2	242	245	Synthesized dsDNA pool			
				3	244	251	1d3, 1d8, 1d10, 1d12, 1f6, 1h2, 1h4, 1h5, Pooled			
7	Segment E randomized 1	1h10, 1f6, 1d3, 1d12	171, 172, 175, 176, 179	1	250	241	1h10, 1f6, 1d3, 1d12 Pooled	8*103		3F, 7f10,7h11, 6b12
				2	242	243				
				3	244	248				
				4	353-362	251	WT			
8	Segment E randomized 2	2c,2f,2i,3F,6b12	171, 172, 175, 176, 179<br>With pool of degenerate NNK primers	1	250	241	2c,2f,2i,3F,6b12<br>Pooled	8*103	3 FACS rounds:<br>Positive after 0.16uM ala, negative after dmso (lower 2%)	5a<br>5b<br>5d
9	Segment E randomized on 5a background	5a	175,176,179	1	250	349	5a	6*1 02	3 FACS rounds:<br>Positive after 50nM ala negative after dmso (lower 2%)	6a
				2	348.1	251				
	Segment E randomized on 5b background	5b	175,176,179	1	250	349	5b	6* 10^{2}		
				2	348.2	251				

Table S3 – List of BdPYL3 variants

identifier	Variations on the wild type BdPYL3 polypeptide sequence
1D3	A97C/F116Y/F171I
1D10	F171H, T172V
1D12	V70I, A97C, F171I
1F6	V89L, V91I, A97C
1H2	F171H, T172V, V176T, N179Q
1H4	F171H, T172V, N179D
1H5	F171H, T172V, V176M, N179D
1H7	F171H, T172V, V176T, N179H
1H10	F116Y, L125S, Y128F
2a	F171H, T172V, N179D
2b	F116Y, S130C, F171H, T172V, N179D
2c	F116Y, L125Y, F171H, T172V, N179D
2f	V70I, A97C, H123N, L125A, F171L
2i	F116Y, F171H, T172V, N179D
2m	V70I, A97C, H123N, S130H, F171L
2q	V70I, A97C, F116H, H123N, F171L
2s	V70I, A97C, H123N, F171L
2t	V70I, A97C, L125S, F171L
3f	V89L, F116Y, S154Y, F171H, T172V
7f10	V89L, F116Y, S154Y, F171H, T172V, T176M, N179D
7h11	G144V, F171H, T172V, V176T, N179Q
6b12	V89L, F171H, T172V, N179F
5a	V70I, A97C, H123N, L125A, F171C, T172C
5b	V70I, A97C, H123N, L125A, F171A, T172I
5c	V70I, A97C, H123N, L125A, S154Y, F171V
5d	V70I, A97C, H123N, L125A, F171I, V176A

Table S4 - Sequences of ssDNA oligos used in library construction

oligo index	name	Sequence 5'-3'
67	(A97C fix) rev	CTCTCTAACAGAACCCACGC
68	(A97C fix) mutagenic fwd	GCGTGGGTTCTGTTAGAGAGSTTACARTTGTGTCTGGTTTGCCTTGTAGCACCAGCACAGAAAGATT
69	reverse for F116Y/L125 fixing mutagenic PCR	ATGACAAAATATGACGATC
70	(F116Y fix) F mutagenic fwd	GATCGTCATATTTTGTCATAYTCTGTAGTAGGTGGTGAAMAYAKGMWKAGGAACTKHAGAAGCGTCACCAGTGTAACAGA
71	(L125S fix) F mutagenic fwd	GATCGTCATATTTTGTCATWYTCTGTAGTAGGTGGTGAAMAYAKGTCGAGGAACTKHAGAAGCGTCACCAGTGTAACAGA
72	(F171H fix) mutagenic fwd - a	GGAAGACGATACAAGGATGCATGKGGATACGKTYRTGAAGTTGVAWTTGCAGAAACTAGCGAGCGT
73	(F171H fix) mutagenic fwd- b	GGAAGACGATACAAGGATGCATRBTGATACGKTYAMYAAGTTGVAWTTGCAGAAACTAGCGAGCGT
240	segment A rev	CTGCTGGAGATGGTGCTAG
241	segment A fwd	CTAGCACCATCTCCAGCAG
242	segmentB rev	TTGGAAATCTTAGATGATGATCGT
243	segment B fwd	ACGATCATCATCTAAGATTTCCAA
244	segment C rev	GAATTTCAGGGACAGGAAGATGC
245	segment C fwd	GCATCTTCCTGTCCCTGAAATTC
248	segement D fwd	CCTTATTGCGTGGTATTG
249	segement D rev	CAATACCACGCAATAAGG
250	BdPYL3 fwd for pBD-GAL4 gibson cloning	AGTTGACTGTATCGCCGGAAATGGAAGCTCACATGGAACG
251	BdPYL3 rev for pBD-GAL4 gibson cloning	GACTCACTATAGGGCTCTAGAGTCGACTTAATCTCTCGTTCTGGAACCA
348.1	segment E mutagenic fwd - 5a mutations fixed	GGAAGACGATACAAGGATGTGTTGTGATACGNNKNNKAAGTTGNNKTTGCAGAAACTAGCGAGCGT
348.2	segment E mutagenic fwd - 5b mutations fixed	GGAAGACGATACAAGGATGGCGATTGATACGNNKNNKAAGTTGNNKTTGCAGAAACTAGCGAGCGT
349	segment E rev	CATCCTTGTATCGTCTTCC
353	segment E mutagenic fwd - 1	GGAAGACGATACAAGGATGNNKNNKGATACGGTTGTAAAGTTGAACTTGCAGAAACTAGCGAGCGT
354	segment E mutagenic fwd - 2	GGAAGACGATACAAGGATGNNKACTGATACGNNKGTAAAGTTGAACTTGCAGAAACTAGCGAGCGT
355	segment E mutagenic fwd - 3	GGAAGACGATACAAGGATGNNKACTGATACGGTTNNKAAGTTGAACTTGCAGAAACTAGCGAGCGT
356	segment E mutagenic fwd - 4	GGAAGACGATACAAGGATGNNKACTGATACGGTTGTAAAGTTGNNKTTGCAGAAACTAGCGAGCGT
357	segment E mutagenic fwd - 5	GGAAGACGATACAAGGATGTTTNNKGATACGNNKGTAAAGTTGAACTTGCAGAAACTAGCGAGCGT
358	segment E mutagenic fwd - 6	GGAAGACGATACAAGGATGTTTNNKGATACGGTTNNKAAGTTGAACTTGCAGAAACTAGCGAGCGT
359	segment E mutagenic fwd - 7	GGAAGACGATACAAGGATGTTTNNKGATACGGTTGTAAAGTTGNNKTTGCAGAAACTAGCGAGCGT
360	segment E mutagenic fwd - 8	GGAAGACGATACAAGGATGTTTACTGATACGNNKNNKAAGTTGAACTTGCAGAAACTAGCGAGCGT

Table S5 – Plasmids constructed and used in this work

Name	Backbone	Description	
pHO-UASg:$_{envy}$GFP:ADH1$_{ter}$	pHO[2]	HO locus targeted integration of *envyGFP*[3] under a GAL4 (Y2H)-regulatable promoter.	
pHO-UASg:*m-Scarlet-I*:ADH1$_{ter}$	pHO	HO locus targeted integration of *m-Scarlet-I*[4] under a GAL4 (Y2H)-regulatable promoter.	
p20StAD	pHO	Cloning and genome integration of a strong expression cassette containing GAL4-AD fused to a gene of interest. ORF of GAL4-AD, multiple cloning site and LEU2 marker are derived from pACT2.<br>Homology arms for site "20" integration[5], TDH3 promoter and TDH1 terminator were amplified from Y190 genomic DNA.	
p20StBD	pHO	Cloning and genome integration of a strong expression cassette containing GAL4-AD fused to a gene of interest. GAL4-BD, multiple cloning site and TRP3 marker are derived from pBD-GAL4.<br>Homology arms for site "21" integration[5], TDH3 promoter and TDH1 terminator were amplified from Y190 genomic DNA.	

1. Menne, H. & Kocher, H. HRAC Classification of Herbicides and Resistance Development. *Mod. Crop Prot. Compd.* **1**, 5–26 (2008).

2. Voth, W. P., Richards, J. D., Shaw, J. M. & Stillman, D. J. Yeast vectors for integration at the HO locus. *Nucleic Acids Res.* **29**, 10–13 (2001).

3. Article, R. Plasmids for C-terminal tagging in Saccharomyces cerevisiae that contain improved GFP proteins, Envy and Ivy. *Yeast* 379–387. doi:10.1002/yea

4. Bindels, D. S. *et al.* MScarlet: A bright monomeric red fluorescent protein for cellular imaging. *Nat. Methods* **14**, 53–56 (2016).

5. Dongmei Bai Flagfeldt, Verena Siewers, Le Huang, and J. N. Characterization of chromosomal integration sites for heterologous gene expression in Saccharomyces cerevisiae. *Yeast* **26**, 545–551 (2009).
